# Integrated profiling identifies CACNG3 as a prognostic biomarker for patients with glioma

**DOI:** 10.1186/s12885-023-10896-1

**Published:** 2023-09-11

**Authors:** Enfang Shan, Yi-nan Cao, Yang Zhang, Wen Chen, Xurui Ren, Shanjie Zhu, Xueru Xi, Shuai Mu, Mian Ma, Tongle Zhi, Xianwen Li

**Affiliations:** 1 School of Nursing, Nanjing Medical University, No.140 Hanzhong Road, Nanjing, Jiangsu Province 210000 China; 2 Department of Medical Imaging, Nanjing Vocational Health School, No. 40, Xiaozhuang, Qixia District, Nanjing, Jiangsu Province 210046 China; 3 Department of Oncology, Senior Department of Oncology, The First Medical Center of Chinese People’s Liberation Army (PLA) General Hospital, Beijing, 100039 China; 4 Department of Neurosurgery, Suzhou Municipal Hospital, Gusu School, The Affiliated Suzhou Hospital of Nanjing Medical University, Nanjing Medical University, No.242 Guangji road, Suzhou, Jiangsu Province 215008 China; 5 The First People’s Hospital of Yancheng, The Fourth Affiliated Hospital of Nantong University, Yancheng, Jiangsu Province 224006 China

**Keywords:** CACNG3, Glioma, Biomarker, Prognostic factor, Overall survival

## Abstract

Gliomas are the most common malignant primary brain tumors in adults with poor prognoses. The purpose of this study is to explore CACNG3 as a prognostic factor that is closely related to the progression and survival outcome of gliomas and to provide a potential new molecular target for the diagnosis and treatment of glioma patients. CACNG3 expression and related clinical data were collected from three major databases of The Chinese Glioma Genome Atlas (CGGA), The Cancer Genome Atlas (TCGA), and Gene Expression Omnibus (GEO). The CGGA dataset was used as a training set, and TCGA and GEO datasets obtained from the GEO database were used for validation. CACNG3 was expressed at low levels in the tumor group, and the overall survival (OS) in patients with low CACNG3 expression is shorter. Furthermore, CACNG3 expression was negatively associated with glioma grades, which was confirmed in the IHC results of clinical samples. The expression level of CACNG3 in the IDH1 wide-type group, 1p/19q non-codel group, and mesenchymal subtype group was significantly reduced, and the results showed that CACNG3 could serve as a biomarker for the mesenchymal molecular subtype. In addition, the univariate and multivariate analysis verified the prognostic value of CACNG3 in predicting the OS of gliomas of all grades. The results of functional annotation and pathway enrichment analysis of differently expressed genes(DEGs), showed that CACNG3 might affect the development of glioma by interfering with synaptic transmission. Moreover, temozolomide (TMZ), commonly used in the treatment of glioma, increased CACNG3 expression in a dose and time-dependent manner. Therefore, CACNG3 plays a vital role in the occurrence and development of gliomas and can serve as a potential biomarker for targeted therapy and further investigation in the future.

**Supplementary Information:**

The online version contains supplementary material available at 10.1186/s12885-023-10896-1.

## Introduction

Gliomas are tumors arising from glial or precursor cells and account for 75% of primary brain tumors in adults [1]. In the WHO 2016 classification system, gliomas are separated into circumscribed gliomas (WHO Grade I) and diffusely infiltrating gliomas (WHO Grade II-IV; whether astrocytic or oligodendroglial) [2]. These tumors vary greatly in histology from benign ependymal tumors to the most aggressive and deadly Grade IV GBM [3]. Malignant gliomas have an unfavorable prognosis and short overall survival time [4]. Conventional therapies, mainly including surgery, radiotherapy, and chemotherapy, have not resulted in major improvements in the survival outcomes of patients with GBM. The optimal diagnosis and treatment of adult gliomas are yet to be discussed [3]. Over the past decade, molecular studies in human tumors have provided important new insights into the complex genetic changes within gliomas that accompany glioma formation and maintenance [5]. The combined phenotypic and genotypic classification of gliomas improves diagnostic objectivity and accuracy, and will probably result in a more precise determination of prognosis and treatment response, to enhance individualized therapeutic plans for patients [1]. The definition of potential biomarkers is particularly important for targeted therapies. Despite the biomarkers already discovered, more reliable biomarkers are needed to guide clinical decisions regarding gliomas [6].

The Gamma3 subunit gene (CACNG3) is a member of the voltage-gated calcium channel (VGCC) gamma subunit gene family (CACNG). Although a good deal is known about the mouse gamma2 gene (CACNG2) and its association with the stargazer mutant mouse line, the role of other gamma subunits is less well-defined [7]. So far, eight members of the subunit CACNG family have been identified. For the most part, the molecular function of the gamma subunits is to downregulate calcium channel activity, of which inappropriate expression or dysfunction gives rise to numerous human neurological disorders, such as Parkinson’s disease, autism, Alzheimer’s disease [8], depression, and especially seizure disorders [9, 10]. It has been shown that CACNG3 is expressed exclusively in the brain [11], located mainly within the postsynaptic densities of the dendritic structures of hippocampal mossy fiber synapses [12]. The distinct role of CACNG3 in channel function and epileptic seizures has been confirmed [13]. However, the role and function of CACNG3 in brain tumors are still unclear.

In this research, we hypothesized that CACNG3 is related to the occurrence and malignancy of gliomas. From this perspective, we conducted further analysis to explore the correlation between CACNG3 expression and clinical parameters to determine its potential mechanisms of mediating glioma progression. Meanwhile, by studying the influence of CACNG3 expression on the overall survival of glioma patients, we assessed its prognostic value in gliomas. Furthermore, we suggested that CACNG3 could serve as a potential biomarker, which may provide novel insights into the diagnosis and treatment of gliomas.

## Materials and methods

### Data extraction and preprocessing

Gene expression data and corresponding clinical data of 325 glioma patients were downloaded from the CGGA website (http://www.cgga.org.cn/). Clinical data collected from the CGGA datasets included grades, IDH1 mutation status, 1p/19q codeletion status (codel or non-codel), gender, age, survival time, and survival status. Different molecular subtypes defined by the TCGA network were also extracted for clinical analysis. Gene expression data and clinical data of a total of 702 patients were obtained from the TCGA website (http://portal.gdc.cancer.gov/). Clinical data collected from the TCGA datasets included grades, IDH1 mutation status, 1p/19q codeletion status (codel or non-codel), survival status, and survival time. CACNG3 gene expression data were analyzed with survival time, survival status, and other clinical traits. The two sets of gene expression were corrected, standardized, and integrated by RStudio (version 4.1.2) and Perl software (version 5.30.2.1). Samples of which CACNG3 expression values were invalid were excluded.

### Human glioma samples

In this study, 12 patients with glioma were randomly selected from The Affiliated Suzhou Hospital of Nanjing Medical University. After the informed consent of the patients, we collected surgically resected glioma samples for analysis. Inclusion criteria: (1) confirmed diagnosis of glioma, in line with the indications of surgical treatment; (2) patients undergoing simple glioma resection; (3) voluntarily participated in the study and signed the informed consent. Exclusion criteria: (1) with a history of cancer; (2) patients with multiple tumors; (3) patients whose postoperative pathology cannot be accurately diagnosed. This study was approved by the Ethics Committee of Nanjing Medical University.

### Immunohistochemistry (IHC)

Paraffin sections of tumor specimens from glioma patients were dewaxed with xylene. After rehydration with gradient alcohol, antigen retrieval was performed with citric acid antigen solution. The sections were sealed in 10% sheep serum and incubated overnight with CACNG3 Polyclonal antibody (Proteintech, Cat No: 13729-1-AP) antibody at 4℃. The corresponding enzyme-conjugate secondary antibody was used to incubate for 1 h at room temperature. DAB was used to incubate for 10 min, and hematoxylin was soaked for 3 min, then washed with PBS and sealed. The staining results were observed under a microscope and the expression differences of tumor tissues were compared.

### Cell culture and drug treatment

U251 cells (Cat Number: KG050) were bought from Jiangsu KeyGEN BioTECH Co (Nanjing, China). U251 cells were cultured in DMEM supplemented with 10% FBS in a humidified environment of 37℃ and 5% CO2. The cells were inoculated on a six-well plate and stimulated with Temozolomide (TMZ). In this study, two variables, TMZ concentration and TMZ administration time, were used for drug treatment. (1) Different concentrations of 0, 6.25, 12.5, 25, 50, and 100 µM were selected. On the second day of plate laying, media containing different concentrations of TMZ were added to each well of the six-well plate. After 24 h of drug stimulation, media containing TMZ was sucked out, cleaned by PBS, photographed, and recorded, and Western blot analysis was performed. (2) When TMZ administration time was used as a variable, 6 administration times of 0, 6, 12, 24, 48, and 72 h were selected. On the second day of plate laying, medium containing 100 µM TMZ was successively added into the corresponding wells according to the duration of administration. After 72 h, the medium containing TMZ was sucked out, cleaned by PBS, photographed, and recorded, and Western blot analysis was performed.

### Western blot analysis

The samples were scraped with a scraper and placed on a shaker for full lysate. After centrifugation, protein concentration was determined with a BCA determination kit and the buffer was added to prepare protein samples. The protein samples were separated and transferred to PVDF membrane with 10% SDS-PAGE, sealed with 5% skim milk at room temperature for 1 h, diluted with CACNG3 (Proteintech, Cat No: 13729-1-AP), Ki67 (Bioworld, Cat No: BS90769), PCNA (Bioworld, Cat No: BS6438), or β-actin (Proteintech, Cat No: 81115-1-RR), and incubated overnight at 4℃. The membranes were incubated with the secondary antibody for 1 h at room temperature. After exposure, protein expression was observed.

### Construction of PPI network and Enrichment analysis of DEGs

The limma package of R language was applied to preliminarily explore the differently expressed genes (DEGs) between patients with high and low CACNG3 expression, in which Fold Change > 2.3 and adj.P < 0.05 were set as the cutoffs to screen for DEGs. Gene Ontology (GO) and Kyoto Encylopedia of Genes and Genomes (KEGG) (www.kegg.jp/kegg/kegg1.html) [14–16] were applied to perform functional annotations of the DEGs and analyze the gene signaling pathways, using ClusterProfiler, org.Hs.eg.db and path view packages of R language. Additionally, the heat map package of R language was used to list the top 30 up-regulated and top 30 down-regulated DEGs. The volcano plot of DEGs was generated to show the up-and-down-regulated genes. The protein-protein interaction (PPI) network was built by uploading the DEGs to the STRING website (https://string-db.org/) and reconstructed by the Cytoscape v3.7.2 software. Then cluster analysis was carried out to retrieve the main gene clusters in the network by using the Molecular Complex Detection (MCODE) plugin. Genes with high combined scores were retained for further GO and KEGG (www.kegg.jp/kegg/kegg1.html) pathway enrichment analysis.

### Correlation analysis

Pearson correlation analysis was used to obtain genes positively associated with CACNG3 expression. The heat map was generated to list the co-expression genes and associated clinical characteristics according to CACNG3 expression. GO and KEGG (www.kegg.jp/kegg/kegg1.html) pathway enrichment analyses were conducted to determine the biological function of the related gene sets.

### Statistical analysis

Kaplan-Meier method followed by a log-rank test was used to compare the survival differences between high level and low levels of CACNG3 expression determined by its median value. The overall survival curve was generated using survival and survminer tools to illustrate the significance of gene expression level of CACNG3 in patients’ survival status. Univariate and multivariate Cox regression analyses were utilized to verify the independent prognostic value of CACNG3 along with associated clinicopathological Characteristics regarding OS (overall survival).

The Boxplot method was applied to visualize the correlation between CACNG3 expression and various clinical traits, in which the Kruskal-Wallis rank-sum test was performed to evaluate the differential expression of CACNG3 among patients with different molecular subtypes defined by the TCGA network and different glioma grades, Wilcoxon rank-sum performed to evaluate the expression difference of CACNG3 among patients with different IDH1 mutation status and 1p/19q deletion status. Samples of which the CACNG3 expression value was missing were excluded. The predictive accuracy of CACNG3 for different molecular subtypes defined by the TCGA network was assessed by receiver operating characteristics (ROC) curves. Calculation results of the area under the curve (AUC) value above 0.8 was considered significant. All statistical analyses and computations conducted in this study were mainly performed using R version 4.1.2.R language packages (ggplot2, heatmap, pROC, and program) were used for other visualization and graphical construction. P < 0.05 was considered the identification of a statistically significant difference.

The results of IHC and WB were presented as the mean ± SD. Statistical comparisons of the different groups were carried out by variance (ANOVA) or Kruskal–Wallis test. ANOVA was used to compare multiple groups. The Kruskal–Wallis test was used for analysis when the data were not normally distributed. Data were analyzed using GraphPrism 8 (GraphPad Software Inc., La Jolla, USA). The level of significance adopted was at most 0.05 for all analyses.

## Results

### CACNG3 is expressed at low levels in the Tumor Group and is related to the overall survival of glioma patients

**Fig. 1:**
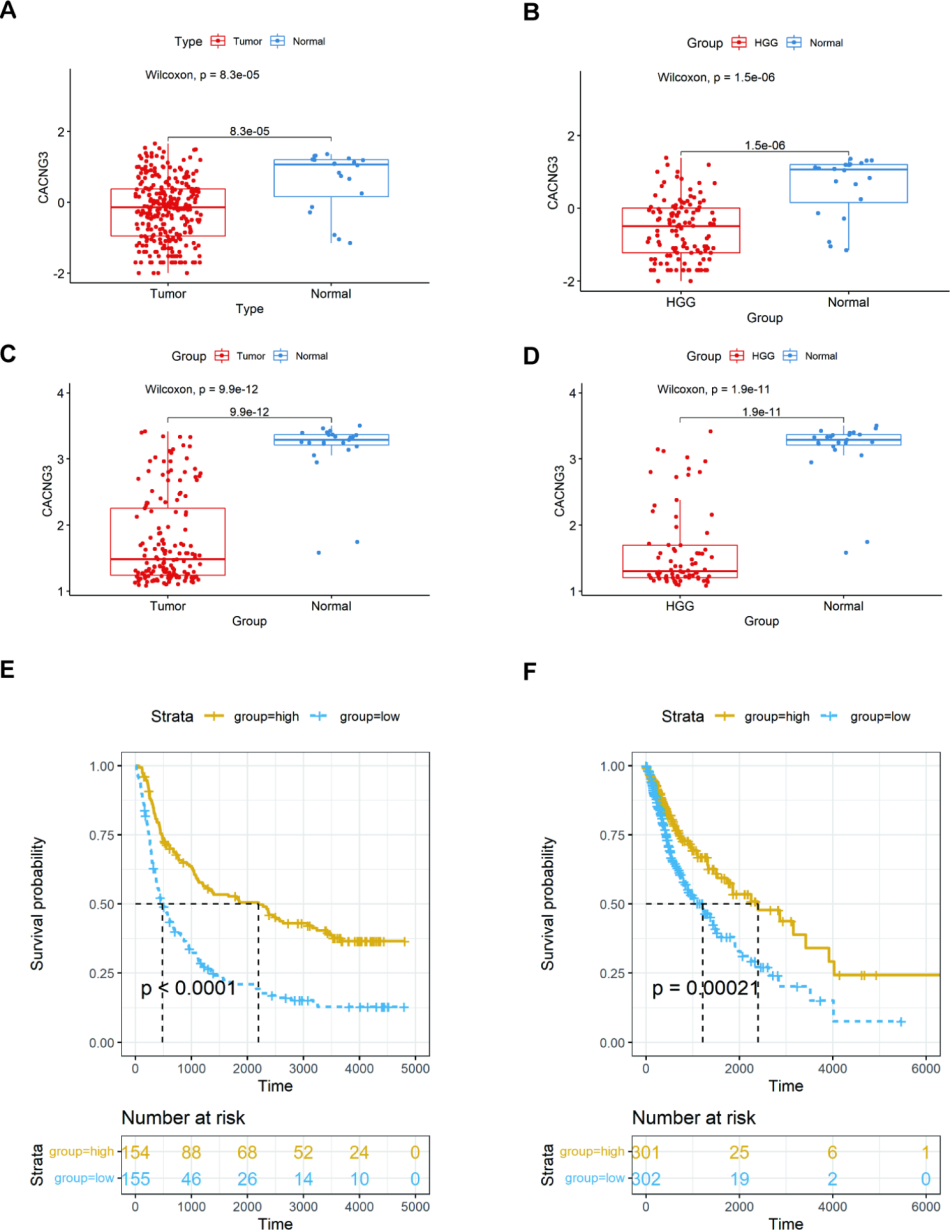
CACNG3 expression correlates with glioma grades. CACNG3 expression decreased in the tumor group, more significantly in the high-grade glioma (HGG) group in CGGA **(A, B)** and GEO4290 datasets **(C, D)**. Different overall survival of all grade glioma patients between high and low CACNG3 expression group. Kanplan-Meier curves showed patients with high CACNG3 expression had longer OS than those with low CACNG3 expression in CGGA **(E)** and TCGA **(F)** datasets

By analyzing gene data of 325 tumor tissue samples and 20 normal tissue samples obtained from the CGGA RNA-seq set and 153 tumor tissue samples and 23 normal tissue samples in GSE 4290 array set, we observed lower expression of CACNG3 in gliomas compared to matched normal tissues, specifically in high-grade gliomas (HGG, WHO Grade IV) (Fig. 1A-D).

Next, we investigated the influence of CACNG3 on the overall survival of glioma patients. Survival data of 325 samples from the CGGA datasets were obtained. Survival data of 603 samples from the TCGA datasets were used for validation. Patients were divided into two groups determined by the different expression levels of CACNG3, using its median value as the cutoff point. Then Kalplan-Meier analysis was conducted to show different survival times and survival statuses of patients in two groups. The results demonstrated that high CANG3 expression led to a remarkably better OS in all grades of gliomas (Fig. 1E, P < 0.0001). The longer survival time of patients with higher CACNG3 expression levels was also validated in the TCGA datasets, which was consistent with previous results (Fig. 1F, P = 0.00021).

### CACNG3 expression is negatively associated with glioma grades

**Fig. 2:**
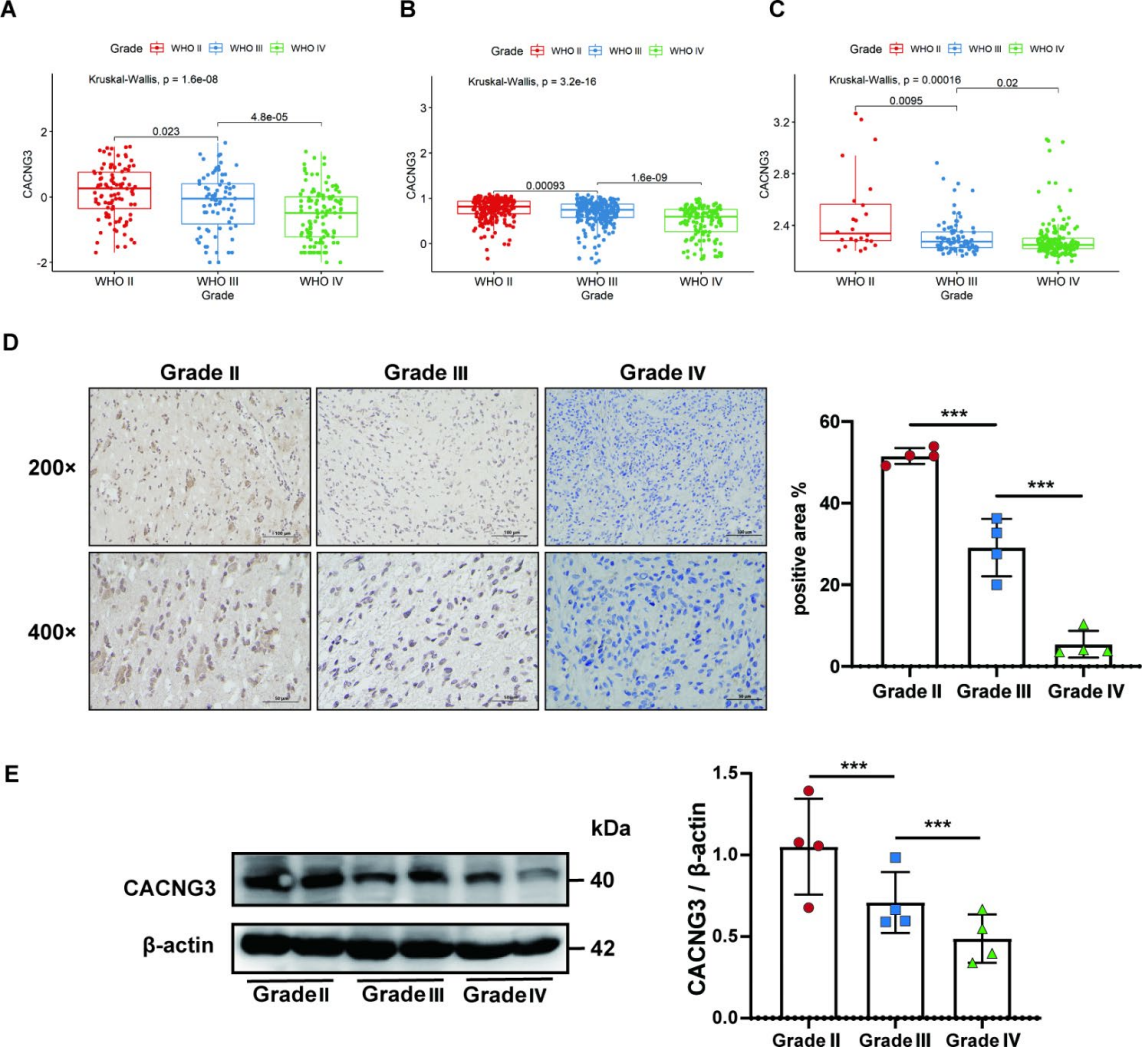
CACNG3 has a lower expression level in higher-grade gliomas in CGGA **(A)**, TCGA **(B)**, and GSE 16,011 datasets **(C)**. CACNG3 expression that was investigated by IHC **(D)** and WB **(E)** decreased in higher-grade gliomas. The blots were cropped, and the full-length blots are presented in Supplementary Fig. 5 for CACNG3 and Supplementary Fig. 6 for β-actin. *** *P* < 0.001 compared with the control group

To further explore the relationship between CACNG3 expression and different glioma grades, clinical data of 325 samples were extracted from the CGGA datasets. The CACNG3 expression in different glioma grades was further analyzed by conducting the Kruskal-Wallis rank-sum test. The results showed that CACNG3 expression was significantly associated with glioma grades, as gene expression decreased in higher-grade gliomas. Similar results were obtained in the TCGA datasets and GSE 16,011 array set (Fig. 2A-C). Furthermore, we obtained glioma samples from The Affiliated Suzhou Hospital of Nanjing Medical University. Both IHC (Fig. 2D) and WB (Fig. 2E) results were consistent with previous results that CACNG3 expression decreased in higher-grade gliomas.

### CACNG3 serves as an independent prognostic factor in Glioma

**Fig. 3:**
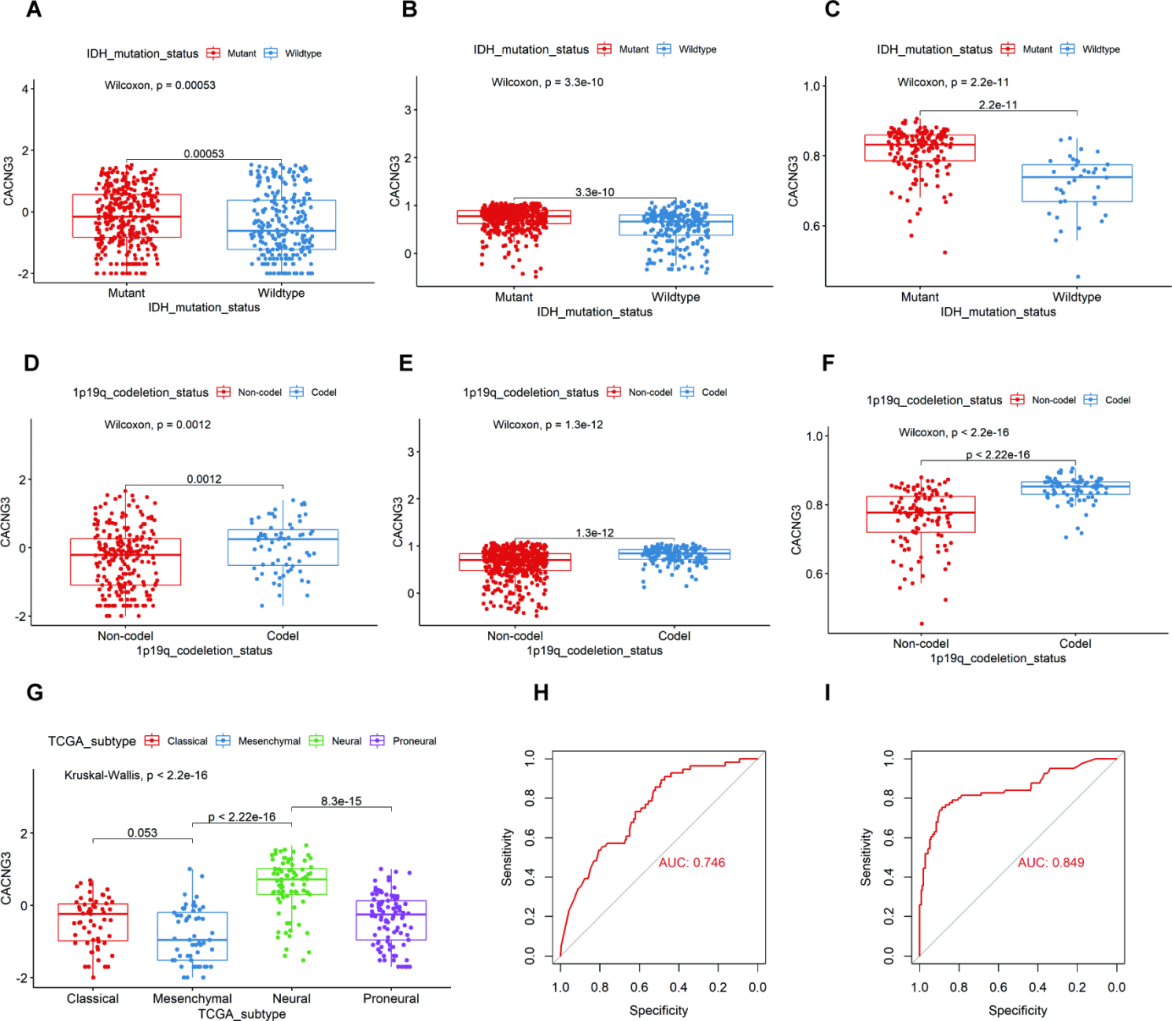
CACNG3 serves as a potential biomarker. CACNG3 expression is associated with IDH mutation status and 1p/19q codeletion status. CACNG3 is enriched in the IDH1 mutation group and 1p/19q codel group in CGGA **(A, D)**, TCGA **(B, E)**, and GSE 58,218 **(C, F)** datasets. Different expression patterns of CACNG3 in four molecular subtypes are defined by the TCGA network. CACNG3 is downregulated in the mesenchymal molecular subtype and significantly upregulated in the neural subtype in CGGA datasets **(G)**. ROC curves of CACNG3 expression to predict mesenchymal and neural subtypes in CGGA datasets **(H, I)**. AUC was calculated and presented

We further studied the expression difference in clinicopathological characteristics of gliomas. Isocitrate dehydrogenase 1 (IDH1) mutations and loss of 1p/19q or co-deletion of both are prognostically significant in glioma patients [17]. IDH mutation status and 1p/19q codeletion status were retrieved from the clinical data downloaded from the CGGA website, and analyzed with the CACNG3 expression data by performing the Wilcoxon rank-sum test. Our results showed that CACNG3 expression was higher in the IDH1 mutation group than in the IDH1 wild-type group (Fig. 3A, P = 0.00053). Increased CACNG3 expression was also observed in the 1p/19q codel group compared with that in the 1p/19q non-codel group (Fig. 3D, P = 0.0012). Fortunately, similar results were obtained by examining CACNG3 expression in different IDH1 mutation status and 1p/19q codeletion status in the TCGA datasets (Fig. 3B and E, *P* < 0.0001) and GEO datasets (Fig. 3 C and F, *P* < 0.0001). The above results indicated that the expression of CACNG3 was associated with a better prognosis and survival outcome in glioma patients.

The transcriptional subtype was an important molecular pathological feature of glioblastoma (GBM). Four transcriptome subtypes (Proneural, Neural, Classical, and Mesenchymal) of GBM were defined by the TCGA network. The Kruskal-Wallis rank-sum test was used to investigate the expression pattern of CACNG3 in different molecular subtypes. The results showed that CACNG3 enriched the neural molecular subtype. Significantly low CACNG3 expression in the mesenchymal molecular subtype was also detected (Fig. 3G, P < 0.0001). As the mesenchymal transition of glioma cells correlates with a more invasive and treatment-resistant phenotype, indicating poor prognosis and recurrence in patients with GBM, down-regulated CACNG3 in the mesenchymal molecular subtype suggests a poor prognosis. To confirm this finding and further explore the predictive ability of CACNG3 as a potential biomarker, we performed the receiver operating characteristic curve (ROC) for CACNG3 expression and both mesenchymal and neural molecular subtypes of all grade gliomas. The area under the curve (AUC) was 74.6% and 84.9% respectively in the CGGA datasets (Fig. 3H and I), which indicated that CACNG3 could serve as a potential biomarker of a mesenchymal and neural molecular subtype of the gliomas, while CACNG3 had a more significant ability in predicting neural molecular subtype.

Next, univariate and multivariate based on the Cox proportional hazards model were carried out in CGGA datasets to evaluate the prognosis value of CACNG3 expression in gliomas of all grades (Table 1). Univariate regression analysis results revealed that CACNG3, together with other clinical characteristics including Age (*P* < 0.0001), IDH mutation status (*P* < 0.0001), 1p/19q codeletion status (*P* < 0.0001), and Grade (*P* < 0.0001) were closely related to patients’ overall survival and could be used to predict OS of gliomas of all grades. The hazard ratio (HR) (commonly used in survival analysis for hypothesis testing) of CACNG3 is less than 1, which represented that the gene expression level was positively related to the survival time of the patients. As for the multivariate regression, CACNG3 showed significant results for predicting 1p/19q codeletion status (*P* < 0.0001), and Grade (*P* < 0.0001). The results of COX analysis suggested that CACNG3 could serve as a prognostic factor in glioma.

**Table 1:** Univariate and multivariate Cox analysis results of CACNG3 and clinical indications in CGGA datasets

Var	HR	CI95.low	CI95.high	P.value	M_HR	M_CI95.low	M_CI95.High	M_P_value
Age	1.03	1.016	1.045	2.30E-05	1.013	0.999	1.027	0.069242
Gender	0.876	0.652	1.176	0.378038	NA	NA	NA	NA
IDH_mutation_status	0.356	0.263	0.482	0	1.007	0.686	1.478	0.970976
Grade	4.592	3.359	6.279	0	2.733	1.913	3.905	0
CACNG3	0.579	0.489	0.686	0	0.696	0.59	0.823	2.00E-05
1p19q_codeletion_status	5.865	3.537	9.723	0	4.038	2.356	6.922	0

### CACNG3 affects the progression of gliomas by involving in the modulation of synaptic transmission

**Fig. 4:**
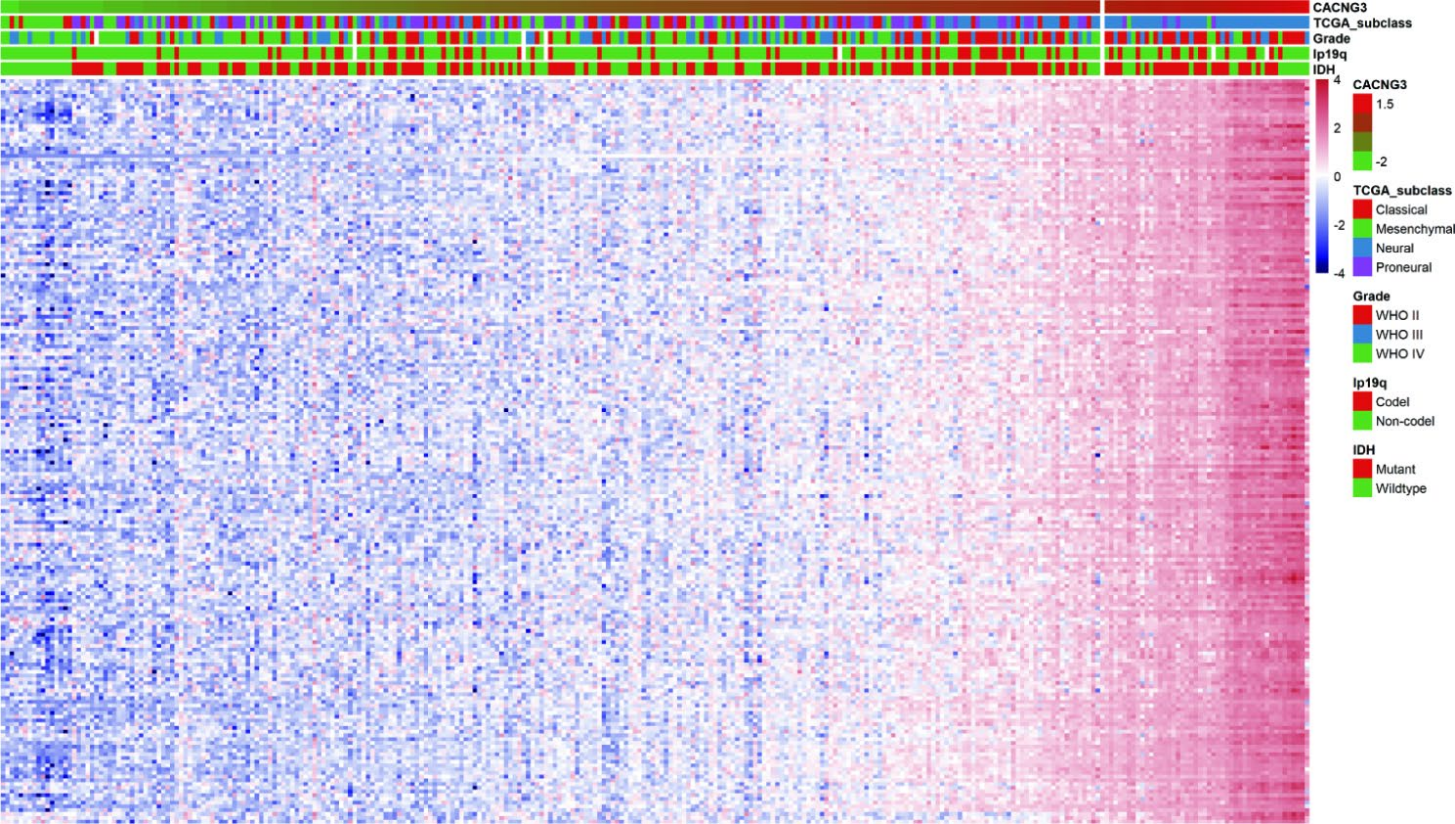
Heat map shows the expression level of 330 genes positively related to CACNG3 expression and clinical information ordered by CACNG3 expression in CGGA datasets

**Fig. 5:**
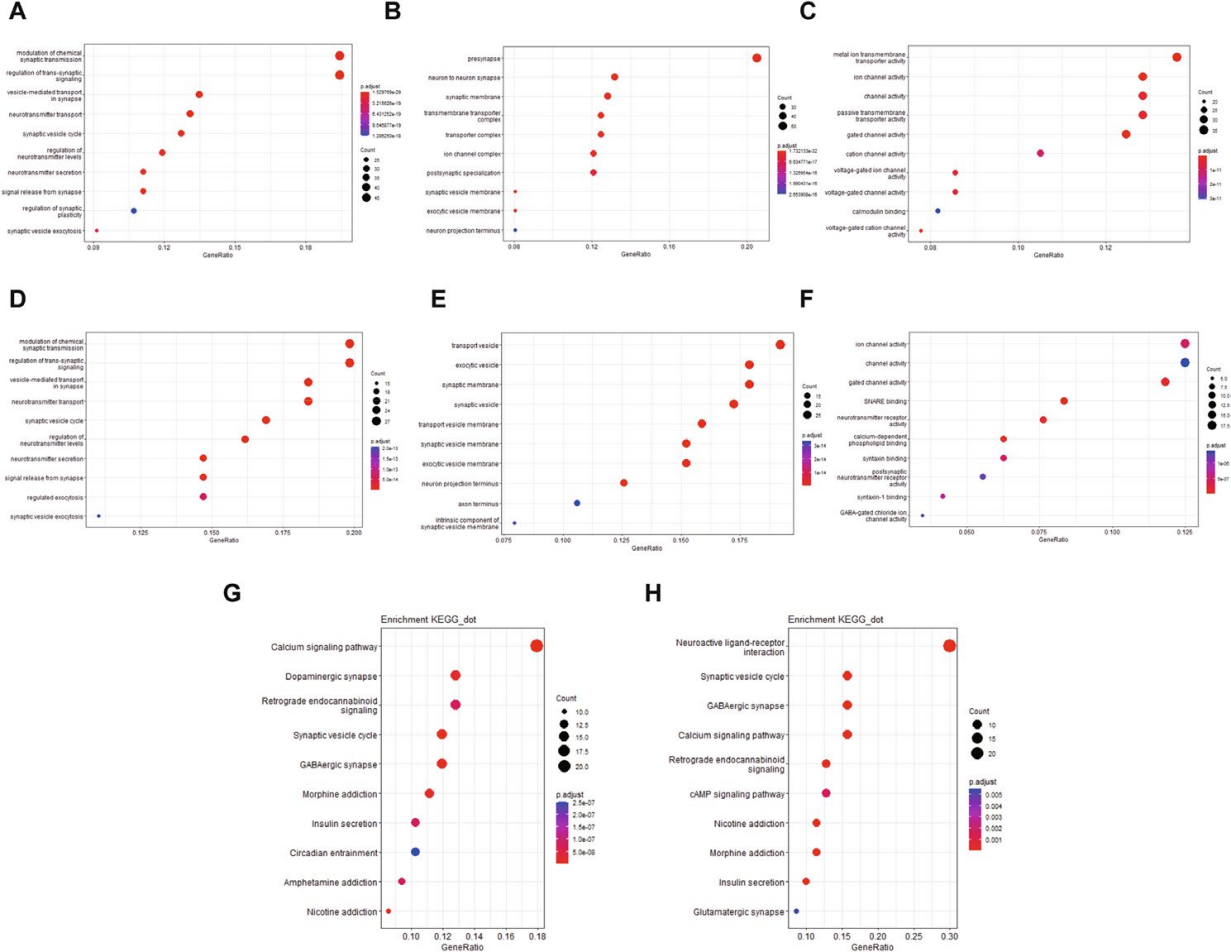
Gene Ontoly (GO) and KEGG (www.kegg.jp/kegg/kegg1.html) pathway enrichment analysis of coexpression genes. The top 10 GO terms in biological process (BP) **(A)**, cellular component (CC) **(B)**, molecular function (MF) **(C)**, and KEGG (www.kegg.jp/kegg/kegg1.html) pathway enrichment analysis results **(D)** based on the CGGA datasets were presented in dot plots, respectively. TCGA database GO enrichment analysis results BP **(E)**, CC **(F)**, MF **(G)**, KEGG (www.kegg.jp/kegg/kegg1.html) pathway analysis results **(H)**. Counts: number of genes in a gene cluster enriched in this GO term or KEGG pathway. p.adjust: adjusted *p*-value of this enrichment result

Given the significant relationship between CACNG3 expression and the malignancy of gliomas, we further analyzed genes positively related to CACNG3 expression by performing the Pearson correlation analysis in the CGGA and TCGA datasets. 330 genes from the CGGA datasets and 172 genes from the TCGA were selected of which the correlation coefficient was greater than 0.8 and the *p*-value was below 0.0001. The heatmap was generated to show the expression level of co-expression genes obtained from the CGGA datasets and clinical pathological information ordered by CACNG3 expression. As is shown in Fig. 4, the positively related genes exhibit a similar expression pattern with CACNG3. In addition, clinical and molecular features, including WHO Grade II and neural subtypes were enriched in gliomas with high CACNG3 expression, which is consistent with previous results. To preliminarily explore the functional characteristics of the co-expression genes, the gene sets were categorized into the biological process (BP), cellular component (CC), and molecular function (MF) functional groups in GO terms. Ten of the GO terms were included in each group. GO analysis results of the CGGA database showed that the co-expression genes were mainly enriched in the presynapse, metal ion transmembrane transporter activity, ion channel activity, modulation of chemical synaptic transmission, and regulation of trans-synaptic signaling (Fig. 5A-C). Based on the KEGG (www.kegg.jp/kegg/kegg1.html) pathway analysis results, these genes were mainly involved in Calcium signaling, Dopaminergic synapse, Retrograde endocannabinoid signaling, Synaptic vesicle cycle, and GABAergic synapse pathway (Fig. 5D). Meanwhile, GO function annotation results from the TCGA database were demonstrated in Fig. 5E-G. KEGG pathway analysis results revealed a remarkable correlation between CACNG3-related genes and Neuroactive ligand-receptor interaction, as is shown in Fig. 5H. All the above results indicate that CACNG3 may affect the glioma cells by modulating synaptic transmission and interfering with particular transmitter signaling pathways.

### Down-regulated DEGs negatively impacted CACNG3 expression and could promote Tumor Invasion

**Fig. 6:**
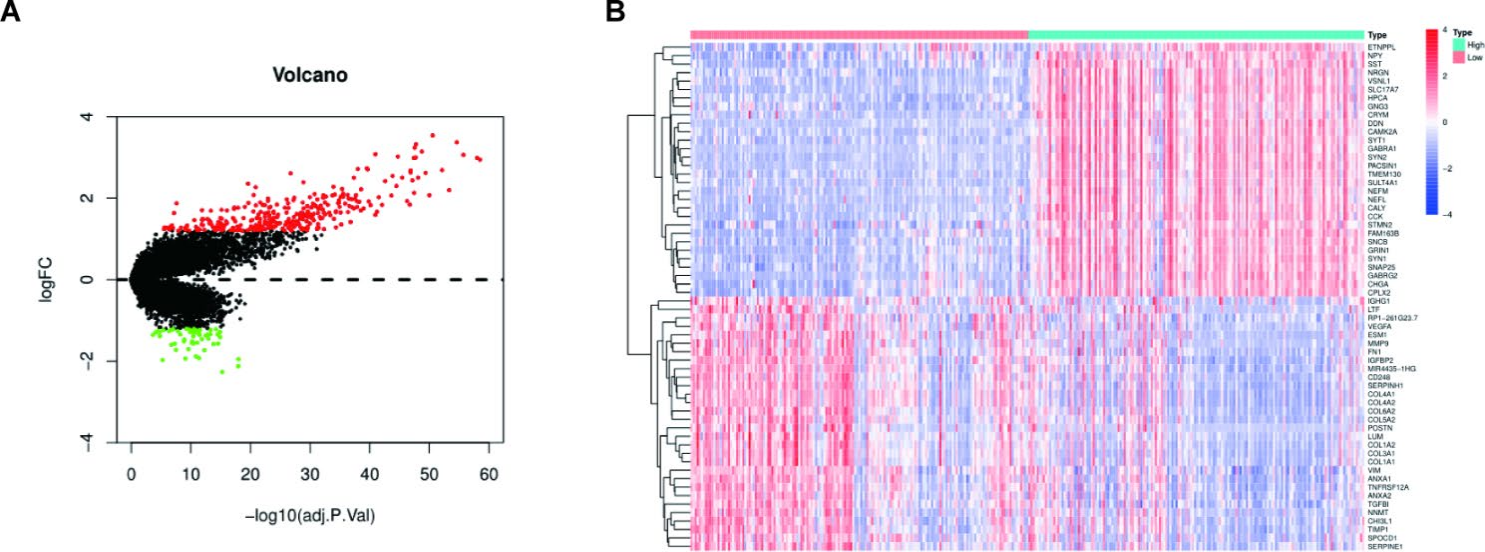
Volcano plot and heat map generated to show the different expression levels of DEGs in high and low CACNG3 expression groups in CGGA datasets. Screening thresholds were set as adj.p.value below 0.05 and log2 fold-change above 1.2. Up-regulated DEGs in the high CACNG3 expression group were presented as red spots and down-regulated DEGs were presented as green spots in the volcano plot **(A)**. The heat map presented the top 30 up- and top 30 down-regulated DEGs **(B)**

Patients with high and low CACNG3 expression were divided into two groups for comparison to screen for DEGs based on CGGA datasets. 307 up-and 56 down-regulated DEGs met the criteria of adj.*P* < 0.05 and Fold Change greater than 2.3 and were presented in the volcano plot (Fig. 6A). The different expression level of the top 30 up- and top 30 down-regulated DEGs in high and low CACNG3 expression group was presented in the heatmap (Fig. 6B). Biologic function analysis of the DEGs would be conducted to further validate our previous findings.

**Fig. 7:**
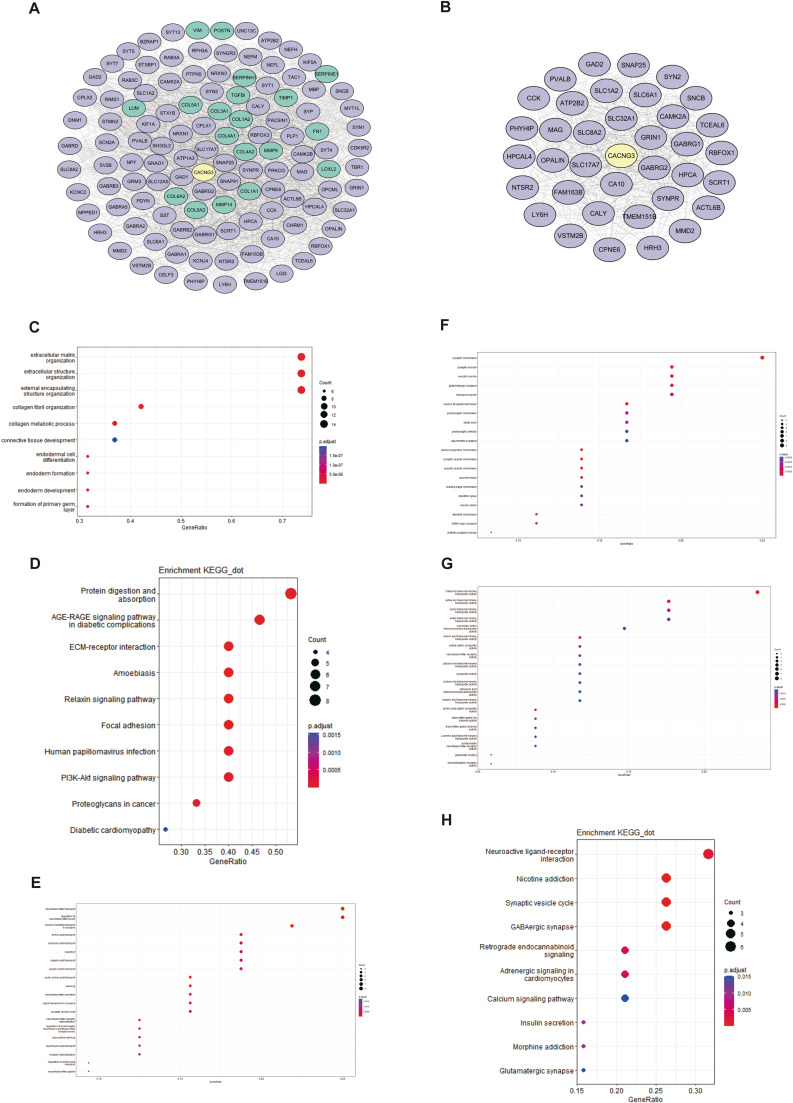
PPI network between CACNG3 and DEGs was constructed, and a biological analysis of the down-regulated DEGs and main gene cluster in the network was conducted. The PPI network was processed with Cytoscape v.3.7.2, of which up-regulated DEGs were colored purple and down-regulated DEGs colored green **(A)**. The main gene cluster was analyzed by MCODE **(B)**. GO terms in BP **(C)** and KEGG (www.kegg.jp/kegg/kegg1.html) pathway analysis results **(D)** of down-regulated DEGs in the network were presented in dot plots. The main gene cluster in GO enrichment analysis results BP **(E)**, CC **(F)**, MF **(G)**, and KEGG (www.kegg.jp/kegg/kegg1.html) pathway enrichment analysis results **(H)**

To understand the interplay among the DEGs positively and negatively associated with CACNG3 expression, a total of 363 DEGs were uploaded to the STRING website to construct a protein-protein interaction (PPI) network. The interaction score was set as 0.4. Cytoscape v3.7.2 software was used to reconstruct the PPI network, and an image with 119 nodes plus 2039 edges was finally obtained (Fig. 7A). The network was made up of 99 genes positively correlated with CACNG3 expression and 19 genes negatively correlated with CACNG3 expression, which indicated that high expression of these genes may either promote or inhibit the expression of CACNG3. Among the 19 down-regulated DEGs, members of typeI and typeIII collagen (COLA) account for a high proportion, which had been reported in previous studies that its expression could be enhanced by the epidermal growth factor (EGF) [18], a protein that activates epidermal growth factor receptor (EGFR) on tumor cells, leading to accelerated tumor growth and poor prognosis [19]. MMP-9, one of the most complex Matrix Metalloproteinase (MMPs), causes the degradation of gelatine and various types of collagen which is essential for tumor invasion and metastasis [20]. Significantly, the majority of the down-regulated DEGs mentioned above were among the top 30 down-regulated genes presented in the heatmap (Fig. 6B). Biological process (BP) and KEGG pathway analysis were performed on the 19 down-regulated DEGs to explore the mechanism by which these genes could have promoted the development of gliomas. The top GO terms in BP comprised extracellular matrix organization and external encapsulating structure organization (Fig. 7C). KEGG pathway analysis results showed that the 19 down-regulated DEGs were mainly involved in Protein digestion and absorption, ECM-receptor interaction, Focal adhesion, and PI3K-AKT signaling pathway (Fig. 7D). The enrichment results suggest that these negatively impacted genes associated with CACNG3 in gliomas may remodel the tumor microenvironment of glioma cells and promote immune escape. Additionally, cluster analysis was carried out to find out the main gene cluster in the current network using MCODE. A total of 37 genes were obtained (Fig. 7B). GO and KEGG pathway enrichment results of these genes indicated that they were mainly involved in the regulation of synaptic transmission, which confirmed the above research conclusions (Fig. 7E-H).

### CACNG3 expression was upregulated by TMZ treatment and CACNG3 overexpression inhibited glioma growth in U251 cells

**Fig. 8:**
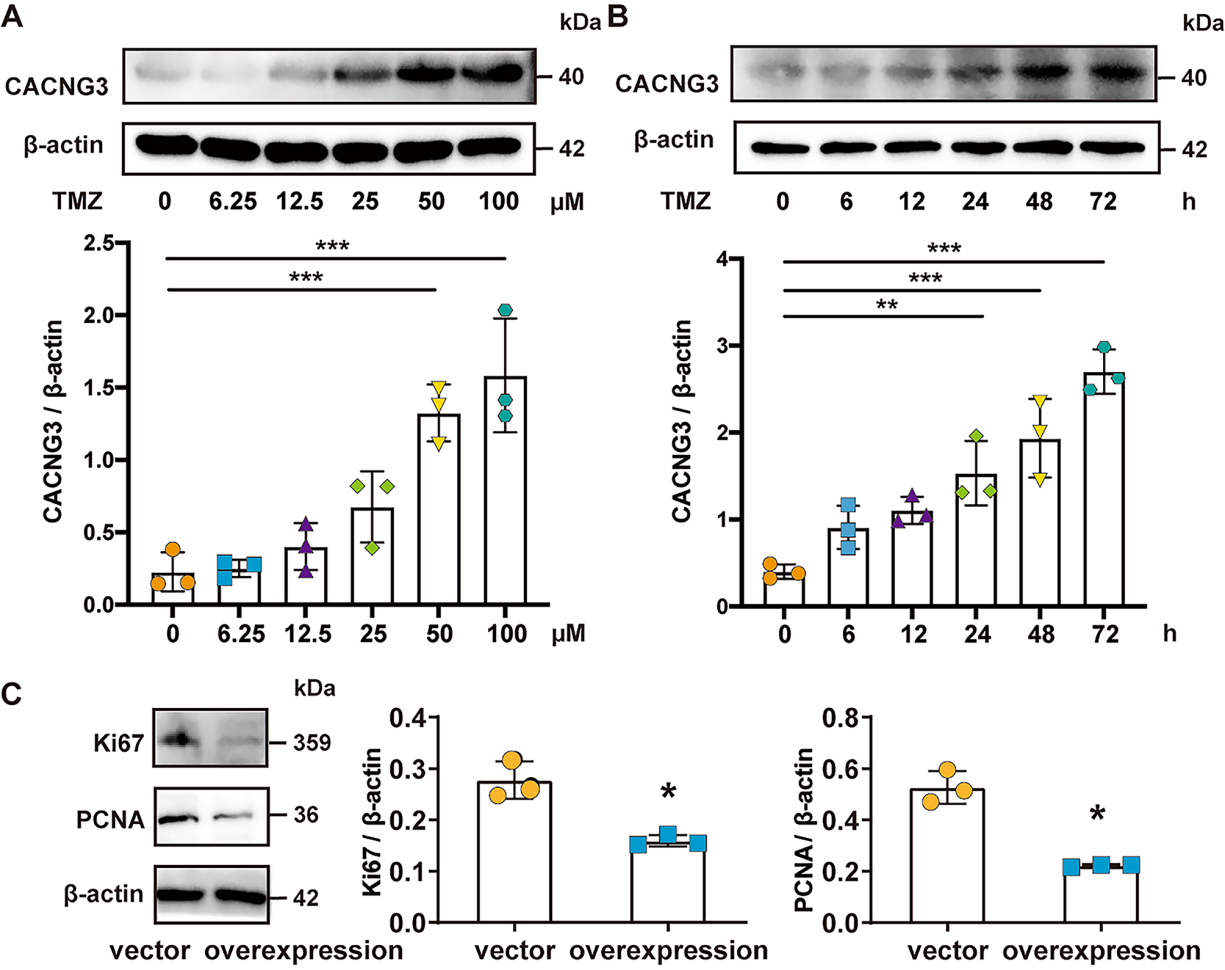
Expression of CACNG3 in U251. **(A)** Western blot was used to detect the expression of CACNG3 in U251 cells treated with TMZ at concentrations of 0, 6.25, 12.5, 25, 50, and 100 µM for 24 h. The blots were cropped, and the full-length blots are presented in Supplementary Fig. 1 for CACNG3 and Supplementary Fig. 2 for β-actin. **(B)** Western blot was used to detect the expression of CACNG3 in U251 cells after TMZ treatment at 0, 6, 12, 24, 48, and 72 h at 100 µM concentration. The blots were cropped, and the full-length blots are presented in Supplementary Fig. 3 for CACNG3 and Supplementary Fig. 4 for β-actin. **(C)** CACNG3 overexpression inhibited Ki67 and PCNA expression. The blots were cropped, and the full-length blots are presented in Supplementary Fig. 7 for Ki67, Supplementary Fig. 8 for PCNA, and Supplementary Fig. 9 for β-actin. The bars showed the means ± SD of three independent experiments. * *P* < 0.05, ** *P* < 0.01 and *** *P* < 0.001 compared with control group

To further observe the expression of CACNG3 in glioma, temozolomide (TMZ) was administered clinically to treat glioma to U251 cells. As shown in the figure, with the increase in TMZ concentration, the expression of CACNG3 in U251 cells showed an increasing trend (Fig. 8A). With the extension of TMZ treatment time, the expression of CACNG3 in U251 cells increased gradually (Fig. 8B). In conclusion, the expression of CACNG3 in U251 cells was positively correlated with the concentration and administration time of TMZ treatment. The results indicated that CACNG3 might play an important role in glioma. To investigate the role of CACNG3 in glioma cells, vector and overexpression plasmid of CACNG3 was trasfected to U251 cells. Interstingly, CACNG3 overexpression decreased the expression of Ki67 and PCNA, the proliferation markers of the tumor, which suggests CACNG3 could inhibit glioma growth (Fig. 8C).

## Discussion

Gliomas are the most common malignant primary brain tumors in adults [1]. Biomarker-targeted therapies could improve treatment efficiencies, as well as guide clinical decisions of gliomas [21]. Over the past decade, molecular genetic alterations have been recognized as more powerful prognoses and predictive markers than histological appearance alone [22]. As a member of the voltage-gated calcium channel gamma subunit gene (CACNG) family, previous studies have reported the role of CACNG3 in downregulating calcium channel activity [23], which associates with numerous neurological disorders, especially seizure disorders [24]. From this perspective, we conducted further analysis to explore the possible role and function of CACNG3 in gliomas. In our study, we preliminarily explored differently expressed genes between the tumor group and the normal group, using gene data extracted from the CGGA RNA-seq set and GSE 4290 array set, and observed significantly lower CACNG3 expression in gliomas (Fig. 1A-D). Furthermore, survival analysis revealed that CACNG3 had a positive influence on the overall survival of glioma patients (Fig. 1E-F).

To determine the correlation between CACNG3 expression and the occurrence and malignancy of gliomas, gene data of glioma patients were obtained from CGGA, TCGA, GSE 16,011, GSE 58,218 datasets and further analyzed with clinical parameters of gliomas (grades, IDH1 mutation status, 1p/19q codeletion status, and different molecular subtypes defined by TCGA network). According to the 2016 WHO classification of gliomas, malignancy and invasiveness increase in higher-grade gliomas, indicating worse prognosis and survival outcome of glioma patients. Our results showed that CACNG3 expression gradually decreased with the increase of glioma grades (Fig. 2A-C). Moreover, IHC results of clinical samples also showed low CACNG3 expression in grade IV gliomas(Fig. 2D). Meanwhile, it has been confirmed in many studies that IDH1 mutations and loss of 1p or 19q or co-deletion of both are of prognostic significance in glioma patients [25]. The results revealed that CACNG3 is up-regulated in IDH1 mutant group (Fig. 3A-C) and down-regulated in the 1p/19q non-codel group (Fig. 3D-F). Besides, the expression level of CACNG3 was significantly raised in the neural subtype group and reduced in the mesenchymal subtype group (Fig. 3G), suggesting that CACNG3 can predict neural and mesenchymal molecular subtype gliomas, while the latter correlates with a more invasive and treatment-resistant phenotype, indicating poor prognosis in glioma patients. In addition, univariate and multivariate analyses indicated that CACNG3 could serve as an independent prognostic factor in gliomas (Table 1). All results discussed above suggested that CACNG3 may play a role in the occurrence and progression of gliomas, as well as predict the survival outcome and treatment response of glioma patients.

By conducting biologic function analyses of 330 genes positively related to CACNG3 expression (Fig. 4), retrieved through correlation analysis using CGGA datasets, we concluded that CACNG3 affected glioma cells by modulating synaptic transmission (Fig. 5). The results were also confirmed in TCGA datasets. Additionally, functional annotation and pathway analysis on 19 DEGs negatively associated with CACNG3 expression were carried out (Fig. 7). The results indicated that these genes could have inhibited the expression of CACNG3 and remodeled the tumor microenvironment of glioma cells, thus promoting the development of glioma cells. Voltage-gated calcium channels (VGCC) are one of the main calcium channels described in cancer cells [26]. It has been reported that calcium channels related to GBM, including VGCC, are involved in the proliferation, invasion, and angiogenesis of glioma cells [27]. Significantly, Zhiqian Zhang’s research group drove liver tumor-initiating cells into apoptosis successfully [28]. Recent studies had also proved an antitumoral effect on human GBM cells by inhibition of VGCC T-type channels, decreasing proliferation migration and growth of GBM cells, finally leading to cell apoptotic death [29]. As is shown in GO function analysis results of co-expression genes of CACNG3, the main molecular function of these genes is to regulate ion channel activity (Fig. 7), and the transmembrane ion flux mediated by ion channels relates to the regulation of tumor progression, especially in gliomas. Several studies have also revealed that voltage-gated ion channels can lead to various processes critical for cancer cell proliferation [23]. Furthermore, derived neurotoxins can induce a temporary disruption of the blood-brain barrier through effective modulation of ion channels, which is arising as a new strategy for brain tumor drug delivery [30]. Surprisingly, alterations in ion channel activity are linked to genetic mutations of tumor suppressors and oncogenes in most brain tumor cases [24], suggesting that up-or down-regulation of CACNG3 may also relate to these encoding genes. Meanwhile, activation of voltage-gated calcium channel channels caused calcium influx which induced glutamate exocytosis [31], a type of amino acid that mediates excitatory, synaptic activations. The glutamate released from gliomas activates glutamatergic receptors in surrounding glioma cells and neurons, increasing calcium influx and further elevating glutamate levels via neuronal release, leading to neuronal death while stimulating the expansion of gliomas [32]. That is to say, glioma cells in both LGG and GBM could actively connect and interact with surrounding cells to facilitate glioma progression. In support, a study published by Nature also demonstrated an AMPA receptor-dependent excitatory postsynaptic current recorded from neuron-to-glioma synapses in over 640 glioma cells [33]. Therefore, as had been concluded in functional annotation results (Fig. 7), CACNG3 may inhibit the development of gliomas by modulating synaptic transmission and other biological processes related to the malignancy of gliomas. However, whether the gamma3 subunit encoded by CACNG3 can interact with glutamate receptors similarly to the gamma2 subunit has yet to be described [34].

Meanwhile, as is shown in the PPI network (Fig. 7A), among the DEGs positively related to CACNG3 expression, 6 members belong to the solute carrier proteins (SLC) superfamily of transporter proteins which enables specific molecules, including anti-tumor drugs, to enter or leave the cell [35]. Thereby, SLCs can affect cancer outcomes by directly affecting the metabolism of tumor cells, as well as indirectly affecting drug pharmacokinetics [36]. Several genes that affect synaptic transmission were also found in the network. For instance, CAMK2A enhances excitatory synaptic transmission and facilitates calcium influx via VGCC [37]through phosphorylation of NMDA [38]and AMPA receptors [39], which is related to Autism spectrum disorder (ASD). SNAP-25 is involved in fast exocytosis in neuronal cells triggered by calcium influx at the synapse [40] and is necessary for postsynaptic NMDA-receptor delivery [41]. Cleavage of SNAP-25 may lead to inappropriate intracellular membrane fusion, and eventually neuronal death [42]. Interestingly, CAMK2A and SNAP25 were among the top 30 up-regulated DEGs and the main gene cluster presented in Figs. 6B and 7B, which suggest a possible interaction between these two genes and CACNG3. Thus, co-expression of these genes may inhibit the occurrence and progression of gliomas mainly through regulatory effects on ion channel activity and synaptic transmission. Further research on the interplay among the DEGs is needed to determine the exact mechanism by which CACNG3 influenced the development of gliomas.

As for cell experiments, temozolomide, a chemotherapeutic drug for glioma, could increase CACNG3 expression in concentration and time dependence (Fig. 8A-B). The results indicated that CACNG3 might play an important role in glioma treatment. To investigate the role of CACNG3 in glioma cells, vector and overexpression plasmid of CACNG3 was trasfected to glioma cells. CACNG3 could inhibit glioma growth, as CACNG3 overexpression decreased the proliferation markers of the tumor (Ki67 and PCNA) (Fig. 8C). Also, to confirm the exact mechanism role of CACNG3 in gliomas, the results of the above studies should be further validated in animals, which will be further explored in our future investigation.

In this study, we analyzed the role and function of CACNG3 in gliomas for the first time and provided a basis for following clinical studies on CACNG3 in gliomas. We assessed the ability of CACNG3 to be a prognostic marker and a biomarker of different glioma molecular subtypes, which may provide novel insights into the diagnosis and treatment of gliomas. Furthermore, CACNG3 may play a critical role in the regulation of synaptic transmission and ion channel activity which inhibits the occurrence and development of gliomas. If CACNG3 can be interfered with in the body, CACNG3 may act as a novel target of glioma therapy. Additionally, in light of the relationship between CACNG3 and the DEGs and the important function of these genes in various cancers, further experiments are needed to explore the interaction between these genes and how they possibly inhibit or promote tumor progression together.

### Electronic supplementary material

Below is the link to the electronic supplementary material.

Supplementary Material 1

Supplementary Material 2

Supplementary Material 3

Supplementary Material 4

Supplementary Material 5

Supplementary Material 6

Supplementary Material 7

Supplementary Material 8

Supplementary Material 9

Supplementary Material 10

## Data Availability

Some data in the study is from public databases, including GEO, CGGA, and TCGA. The direct links are as follows. For GEO: GSE4290: https://www.ncbi.nlm.nih.gov/geo/query/acc.cgi?acc=GPL570. https://ftp.ncbi.nlm.nih.gov/geo/series/GSE4nnn/GSE4290/matrix/. GSE16011: https://www.ncbi.nlm.nih.gov/geo/query/acc.cgi?acc=GPL8542. https://ftp.ncbi.nlm.nih.gov/geo/series/GSE16nnn/GSE16011/matrix/. GSE58218: https://www.ncbi.nlm.nih.gov/geo/query/acc.cgi?acc=GPL13534. https://ftp.ncbi.nlm.nih.gov/geo/series/GSE58nnn/GSE58218/matrix/. For CGGA: http://www.cgga.org.cn/download.jsp#mRNAseq_693. http://www.cgga.org.cn/download.jsp#mRNAseq_325. For TCGA: http://www.cgga.org.cn/download_other.jsp.

## Abbreviation

AUC: Area under the curve
CACNG3: Gamma3 subunit gene
CGGA: Chinese Glioma Genome Atlas
DEGs: Differently expressed genes
GEO: Gene Expression Omnibus
GO: Gene Ontology
HR: Hazard ratio
IDH1: Isocitrate dehydrogenase 1
KEGG: Kyoto Encyclopedia of Genes and Genomes
MCODE: Molecular Complex Detection
OS: Overall survival
PPI: Protein-protein interaction
ROC: Receiver operating characteristic curve
TCGA: The Cancer Genome Atlas
TMZ: Temozolomide
