# Supplementary material for: Integrated profiling identifies CACNG3 as a prognostic biomarker for patients with glioma
Source: BMC Cancer. 2023 Sep 11;23:846. doi: 10.1186/s12885-023-10896-1 (PMC10494363; doi:10.1186/s12885-023-10896-1)
Supplement: Supplementary file 1 — Supplementary Material 1 [file 12885_2023_10896_MOESM1_ESM.docx]

**Supplementary Figure Legends**

**Supplementary Figure 1 |** Original of Figure 8A. The full-length blot for CACNG3 is presented in concentration-dependence.

**Supplementary Figure 2 |** Original of Figure 8A. The full-length blot for β-actin (concentration-dependence experiments) is presented.

**Supplementary Figure 3 |** Original of Figure 8B. The full-length blot for CACNG3 is presented in time dependence.

**Supplementary Figure 4 |** Original of Figure 8B. The full-length blot for β-actin (time-dependence experiments) is presented.

**Supplementary Figure 5 |** Original of Figure 2E. The full-length blot for CACNG3 in different grades of glioma samples is presented.

**Supplementary Figure 6 |** Original of Figure 2E. The full-length blot for β-actin in different grades of glioma samples is presented.

**Supplementary Figure 7 |** Original of Figure 8C. The full-length blot for Ki67 is presented in the CACNG3 overexpression experiment.

**Supplementary Figure 8 |** Original of Figure 8C. The full-length blot for PCNA is presented in the CACNG3 overexpression experiment.

**Supplementary Figure 9 |** Original of Figure 8C. The full-length blot for β-actin is presented in the CACNG3 overexpression experiment.
