# Supplementary figures and images for: Integrated profiling identifies CACNG3 as a prognostic biomarker for patients with glioma
Source: BMC Cancer. 2023 Sep 11;23:846. doi: 10.1186/s12885-023-10896-1 (PMC10494363; doi:10.1186/s12885-023-10896-1)

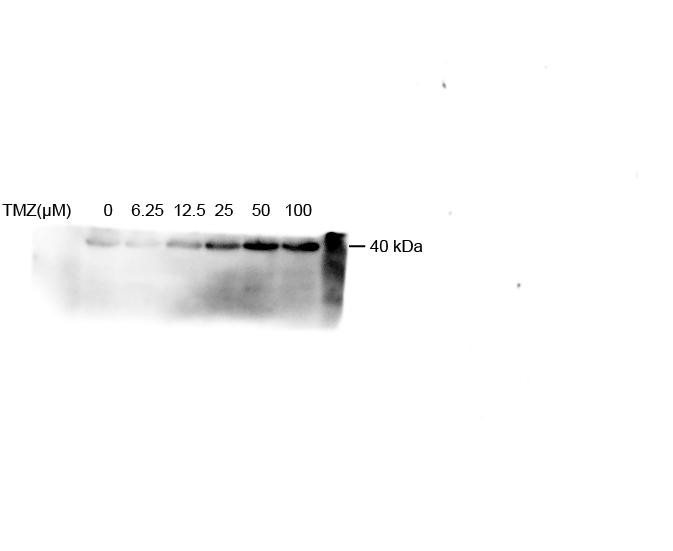

Supplement: Supplementary file 2 — Supplementary Material 2 [file 12885_2023_10896_MOESM2_ESM.jpg]

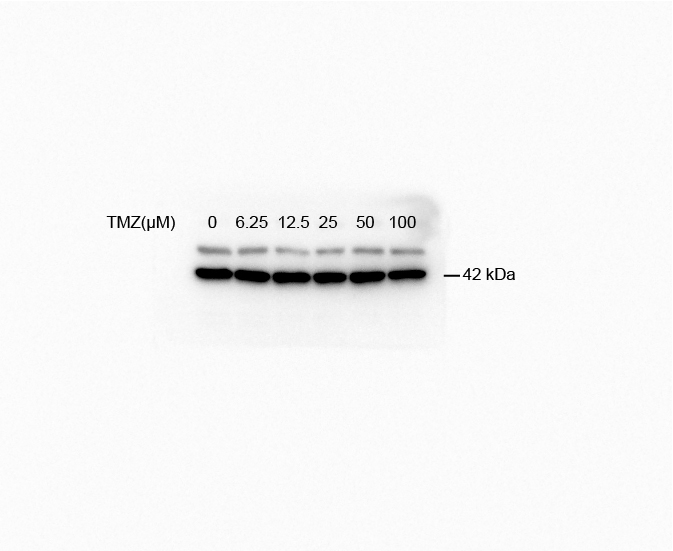

Supplement: Supplementary file 3 — Supplementary Material 3 [file 12885_2023_10896_MOESM3_ESM.jpg]

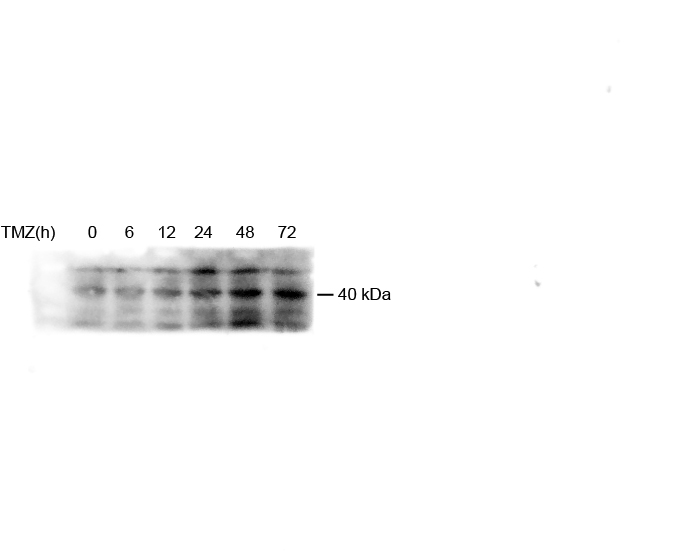

Supplement: Supplementary file 4 — Supplementary Material 4 [file 12885_2023_10896_MOESM4_ESM.jpg]

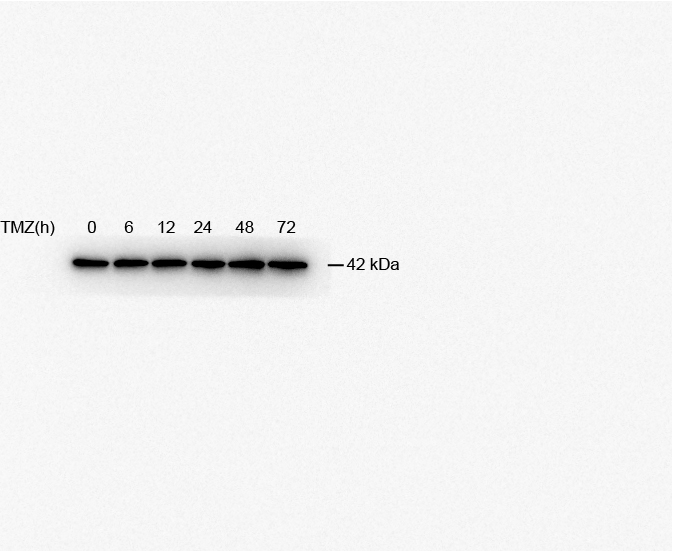

Supplement: Supplementary file 5 — Supplementary Material 5 [file 12885_2023_10896_MOESM5_ESM.jpg]

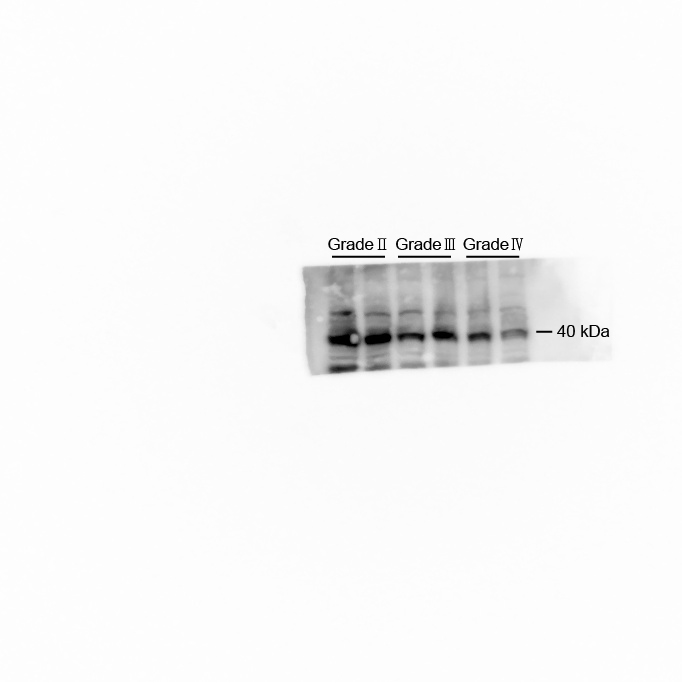

Supplement: Supplementary file 6 — Supplementary Material 6 [file 12885_2023_10896_MOESM6_ESM.jpg]

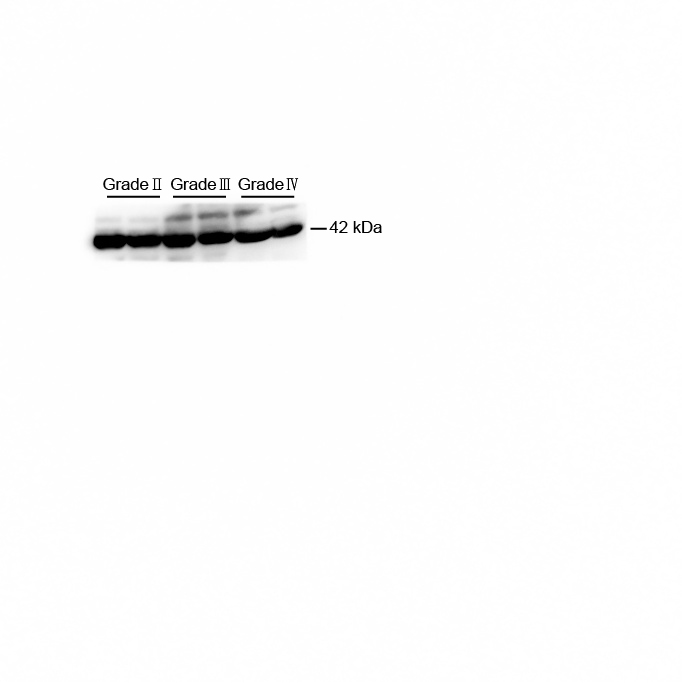

Supplement: Supplementary file 7 — Supplementary Material 7 [file 12885_2023_10896_MOESM7_ESM.jpg]

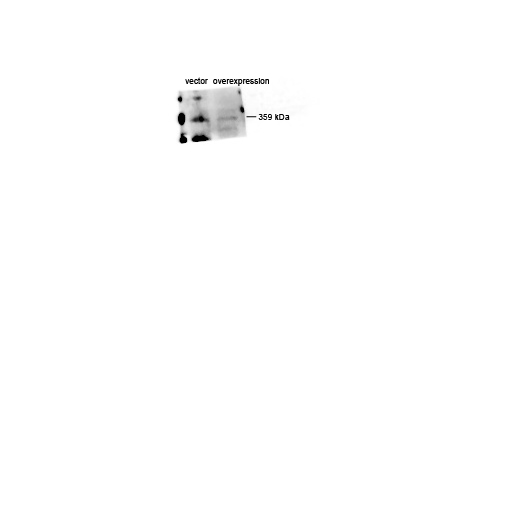

Supplement: Supplementary file 8 — Supplementary Material 8 [file 12885_2023_10896_MOESM8_ESM.jpg]

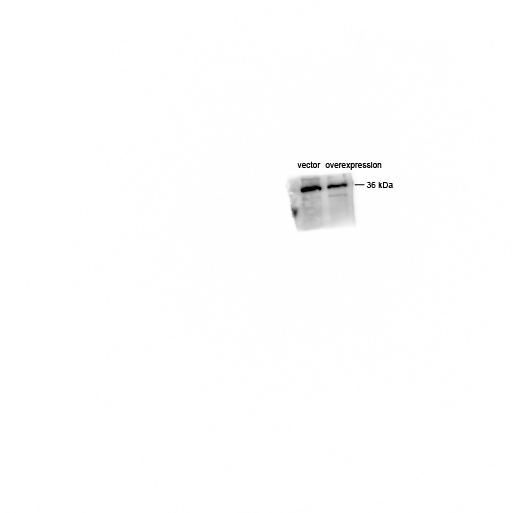

Supplement: Supplementary file 9 — Supplementary Material 9 [file 12885_2023_10896_MOESM9_ESM.jpg]

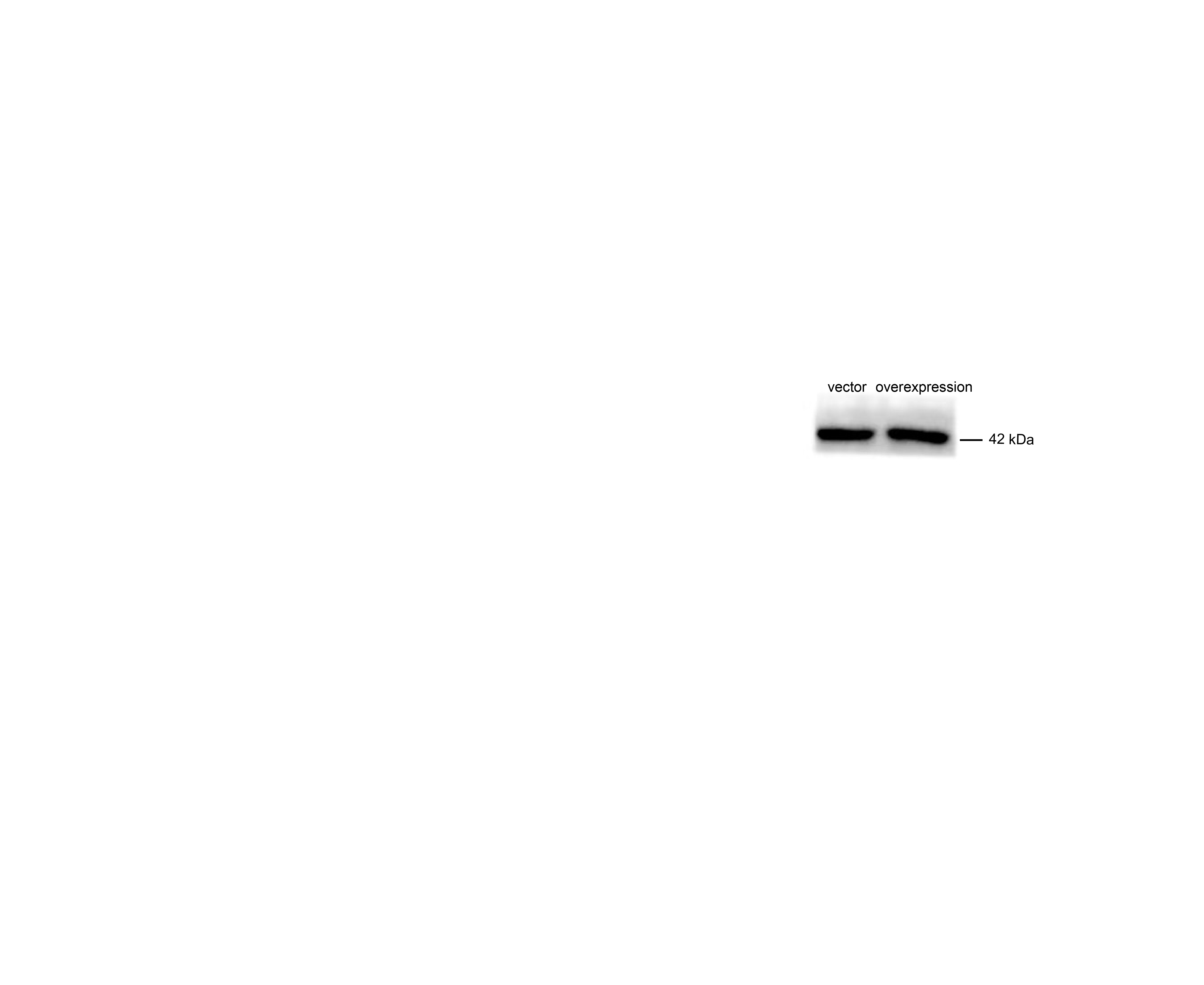

Supplement: Supplementary file 10 — Supplementary Material 10 [file 12885_2023_10896_MOESM10_ESM.jpg]
